# Impact of synthetic colloids on organ function in patients with severe sepsis

**DOI:** 10.1186/cc9504

**Published:** 2011-03-11

**Authors:** O Bayer, M Kohl, B Kabisch, N Riedemann, Y Sakr, C Hartog, K Reinhart

**Affiliations:** 1 Friedrich-Schiller-University, Jena, Germany

## Introduction

Previous studies showed an increased risk for developing acute kidney injury in septic patients receiving synthetic colloids [1]. However, little is known about effects of synthetic colloids on other organs. Ginz and colleagues found altered organ morphology and considerable colloidal storage in parenchymal and reticuloendothelial cells of the liver, lung and kidney in a septic patient after synthetic colloid administration [2]. For this reason we analyzed the effects of HES and gelatin on kidney, liver and lung function in comparison with crystalloids in septic patients.

## Methods

A prospective controlled before-and-after study in 1,046 patients with severe sepsis. Acute kidney injury (AKI) was defined by RIFLE criteria and/or by new occurrence of renal replacement therapy (RRT). Liver function was determined by aspartate aminotransferase (AST), alanine aminotransferase (ALT), bilirubin blood levels during the first 14 days, lung function by PaO_2_/FiO_2 _ratio and ventilation time. Between 2004 and 2006, standard colloid was HES (mainly 6% HES 130/0.4 (87%) and 10% HES 200/0.5). Between 2006 and 2008, standard colloid was changed to 4% gelatin (Gel). From 2008 until April 2010, patients received only crystalloids (Crys).

## Results

Groups were comparable at baseline concerning SAPS II and SOFA scores, age and renal function. Patients who received synthetic colloids more often met the criteria for AKI (Crys 58.4%, HES 70.6% *P *= 0.001, Gel 67.6% *P *= 0.012) or required RRT (Crys 27.8%, HES 34.2% *P *= 0.072, Gel 35.5% *P *= 0.031) than patients receiving only crystalloids. On day 3, liver enzymes peaked in both colloid groups but not in the crystalloid group (AST (μmol/l), median (IQR): HES 2.2 (0.9 to 6.3) *P *= 0.001, Gel 1.7 (0.7 to 3.7) *P *= 0.158, Crys 1.0 (0.6 to 3.2); ALT: HES 1.1 (0.5 to 3.1) *P *= 0.003, Gel 0.9 (0.4 to 2.1) *P *= 0.109, Crys 0.6 (0.3 to 1.9). Bilirubin levels remained significantly elevated from day 0 to 14 in the HES and Gel groups. Median ventilation time (hours) was significantly longer in the HES and Gel groups: HES 214 (60 to 368) *P *< 0.001, Gel 146 (48 to 333) *P *= 0.002, Crys 105 (15 to 280). The PaO_2_/FiO_2 _ratio and ICU or hospital mortality did not show significant differences.

## Conclusions

HES and gelatin may be associated with an increased risk of renal failure, impaired liver function and longer ventilation time in septic patients.

## References

[B1] Brunkhorst N Engl J Med 2008 358 125 139 10.1056/NEJMoa070716 18184958

[B2] Ginz Anaesthesist 1998 47 330 334 10.1007/s001010050564 9615850

